# Perirhinal and Postrhinal Damage Have Different Consequences on Attention as Assessed in the Five-Choice Serial Reaction Time Task

**DOI:** 10.1523/ENEURO.0210-21.2021

**Published:** 2021-09-22

**Authors:** Sean G. Trettel, Kara L. Agster, Rebecca D. Burwell

**Affiliations:** 1 Department of Cognitive, Linguistic, and Psychological Science, Brown University, Providence, RI, 02912; 2 Department of Neuroscience, Brown University, Providence, RI, 02912

**Keywords:** executive function, hippocampus, medial temporal lobe, memory, parahippocampal, serial reaction time

## Abstract

The perirhinal (PER) and postrhinal (POR) cortices, structures in the medial temporal lobe, are implicated in learning and memory. The PER is understood to process object information and the POR to process spatial or contextual information. Whether the medial temporal lobe is dedicated to memory, however, is under debate. In this study, we addressed the hypothesis that the PER and POR are also involved in non-mnemonic cognitive functions. Rats with PER or POR damage and SHAM surgical controls were shaped, trained, and tested on the five-choice serial reaction time (5CSRT) task, which assesses attention and executive function. Rats with PER damage were impaired in acquiring the task and at asymptote, although processing information about objects was not relevant to the task. When confronted with attentional challenges, rats with PER damage showed a pattern consistent with decreased attentional capacity, increased response errors, and increased impulsive behavior. Rats with POR damage showed intact acquisition and normal asymptotic performance. They also exhibited faster latencies in the absence of speed accuracy trade-off suggesting enhanced response readiness. We suggest this increased response readiness results from decreased automatic monitoring of the local environment, which might normally compete with response readiness. Our findings are consistent with a role for PER in controlled attention and a role for POR in stimulus-driven attention providing evidence that the PER and POR cortices have functions that go beyond memory for objects and memory for scenes and contexts, respectively. These findings provide new evidence for functional specialization in the medial temporal lobe.

## Significance Statement

The perirhinal (PER) and postrhinal (POR) cortices, structures in the medial temporal lobe, are implicated in learning and memory. Whether medial temporal lobe structures are exclusively dedicated to memory, however, is under debate. We provide evidence for a role for PER in controlled attention and a role for POR in stimulus-driven attention. These findings provide new evidence for functional specialization in the medial temporal lobe.

## Introduction

Episodic memory, or memory for the everyday events of life, is understood to be supported by the medial temporal lobe (Squire et al., 2004; Eichenbaum et al., 2007). In primates, the medial temporal lobe comprises the hippocampal formation and the nearby structures of the parahippocampal region including the perirhinal cortex (PER) and the parahippocampal cortices, the postrhinal cortex (POR) in the rodent brain (Burwell et al., 1995; Beaudin et al., 2013). The PER is thought to process object information and the POR to process spatial or contextual information in the service of episodic memory.

Whether all medial temporal lobe structures are dedicated exclusively to mnemonic functions remains under debate. A number of studies have provided evidence that different structures in the medial temporal lobe make different contributions to memory and learning (Bussey et al., 1999; Jarrard et al., 2004; Eacott and Gaffan, 2005; Eichenbaum et al., 2007; Ramos, 2013; Heimer-McGinn et al., 2017). There is evidence for functional specialization along the septotemporal axis (Maurer and Nadel, 2021). Some investigators have proposed that components of the medial temporal lobe memory system are differentiated by what is represented and how information is processed (Jarrard et al., 2004; Bussey and Saksida, 2007; Lawrence et al., 2020). Finally, there is evidence that some medial temporal lobe structures have non-mnemonic functions including perception (Bucci and Burwell, 2004; Bussey and Saksida, 2007; Ahn and Lee, 2017; Gaynor et al., 2018). We previously provided evidence that the POR has a role in attention (Burwell and Hafeman, 2003; Bucci and Burwell, 2004). In this study, we addressed the hypothesis that PER and POR support different aspects of attention in the service of learning and memory as well as other cognitive processes.

We have proposed that the POR represents context by combining spatial information with information about discrete items and objects provided by the PER (Furtak et al., 2012; Heimer-McGinn et al., 2017). We further proposed that the POR is involved in ongoing and automatic monitoring of the context for changes (Burwell and Hafeman, 2003). In contrast, the PER is involved in processing individual objects and items, especially when such stimuli are complex or ambiguous (Cowell et al., 2010). Such findings suggest the POR and PER may be involved in different sorts of attention, i.e., stimulus driven and controlled attention, respectively.

In the present study, we used the five-choice serial reaction time (5CSRT) task to address the hypothesis that the PER and POR functions include different types of attention. The 5CSRT task is a powerful tool for assessing multiple dimensions of attention, including controlled, sustained, divided, selective, and visuospatial attention (Carli et al., 1983; Winstanley et al., 2003; Bari et al., 2008). Rats with damage to either PER or POR were shaped and trained to asymptote on the task. They were then presented with a series of attentional challenge conditions including noise distractors, shortened stimulus durations, variable intertrial intervals (ITIs), and a random tone distractor.

## Materials and Methods

### Subjects

Subjects were 28 male Long–Evans rats (Charles River Laboratories) weighing between 250–300 g at the start of the experiments. Animals were allowed a few days to adjust to the animal colony. At that point, we began handling the animals daily. After approximately one week, the animals were put on a food schedule such that one meal a day was provided with the goal of maintaining body weight at 85–90% of the free feeding weight at the same age. Water was freely available throughout the experiment. Animals were maintained on a 12/12 h reverse light/dark cycle. Thus, all testing occurred during the dark period of the light cycle. This research was conducted according to Brown institutional and federal animal care and use guidelines.

### Surgery

Animals were placed in an anesthesia induction chamber and lightly anesthetized with isoflurane gas. The scalp was shaved and subjects were placed in a stereotaxic apparatus (David Kopf Instruments). All surgical procedures were conducted under isoflurane anesthesia. Animals were also given glycopyrrolate (0.25 mg/kg, i.p.) to reduce the occurrence of respiratory difficulties. Once fully anesthetized an incision was made over the top of the skull; the skin and fascia were retracted. The skull overlying the POR or PER was removed. A microinjection unit (David Kopf Instruments) was used to deliver either a quantity of ibotenic acid (lesion cases) or sterile saline (sham controls). A volume of 50 nl of ibotenic acid (10 μg/1 μl) was injected to each site in the PER and a volume of 75 nl was injected to each site in the POR. The micropipette was lowered to the appropriate coordinate and allowed to sit for 1 min; following delivery of the excitotoxin or saline, the micropipette was left in place for 2 min and then slowly raised and removed from the brain. After all injections were made the skull was cleaned and the wound sutured. Animals were then returned to their home cage and placed under a warming light. Once active, subjects were given a dose of rimadyl (Bio-Serv, 6 mg, per os) to relieve pain. Animals were then returned to the colony, and observed for 3 d to ensure proper healing of the wound. Subjects were allowed to recover for a period of two weeks before starting behavioral testing. Table 1 shows the lesion coordinates for PER lesioned subjects (*n* = 8) and POR lesioned subjects (*n* = 8). One half of sham controls (*n* = 6) received saline injections at PER locations, and the other half (*n* = 6) received saline injections at POR locations.

**Table 1:** Lesion coordinates

Region	AP	ML	DV
PER	−3.3	5.7	−6.7
	−4.3	5.7	−6.7
	−5.3	5.7	−6.7
	−6.3	5.7	−6.7
	−7.4	5.7	−6.2
POR	0.54	5.3	−5.5
	−0.46	5.3	−5.5
	−1.46	5.3	−5.5

Perirhinal anteroposterior (AP) coordinates are measured in mm from bregma. Postrhinal AP coordinates are measured in mm from λ. Mediolateral (ML) coordinates are measured in mm to the left and to the right from the midline. Dorsoventral (DV) coordinates are measured in mm from skull.

### Histology

At the completion of the experiment animals were given a lethal overdose of Beuthanasia (Schering-Plough) and transcardially perfused. First, normal saline was perfused to clear the blood. Subsequently, 10% formalin was perfused to fix the tissue. Subjects were decapitated and brains were removed for histologic processing. Brains were cryoprotected in a 20% glycerin solution and subsequently sectioned on a freezing microtome. We collected a 1:2 series of coronal sections with a thickness of 40 μm. Sections were mounted on gelatin subbed slides and allowed to dry overnight in an oven (40°C)*. S*lides were defatted for 60 min in a 50% ethanol/50% chloroform solution, rehydrated through a descending series of alcohols to rehydrate the tissue (100%, 95%, 75%, and 50% ethanol), stained with thionin solution, dehydrated through an ascending series of alcohols to dehydrate the sections, placed in a xylenes series, and coverslipped.

Lesions were quantified using the Cavalieri method as in earlier reports (Heimer-McGinn et al., 2017). Briefly, every other 40-μm section was examined at 4× magnification and regions of interest were drawn. Regional borders were marked off and damaged areas were highlighted. Damaged tissue both inside and outside the region of interest was quantified. Tissue that exhibited cell death, damage, or cortical thinning was considered functionally damaged. For each coronal section, we quantified the total area of the target region, the area of the target region that was damaged, and the area that was damaged in non-target regions for the left and right hemispheres. We then calculated the percent of the target area that was damaged, and the percent of the coronal sections that exhibited any amount of damage. In earlier studies, we have found that the rostrocaudal extent of the damage is a better predictor of lesion effect than area or volume. For that reason, subjects were retained in the study if the lesion was distributed along the rostrocaudal axis in both hemispheres. In addition, subjects with bilateral damage to an extra-target region were eliminated from the study.

### Apparatus

Behavioral training was conducted in an operant testing environment controlled by the MED-PC software package (Med Associates). Custom software written in the Pascal-based, MED-PC notation controlled the behavioral tasks and recorded task events and responses. Experiments were conducted in six 24.0 × 30.5 × 29.0 cm operant test chambers with aluminum panels in the front and back, Plexiglas side walls and top, and a grid floor. A dimly illuminated food cup was recessed in the center of one end wall. A photograph beam mounted inside the recessed food cup permitted automated assessment of food-cup behavior. The opposite wall was curved and incorporated 5 nose poke holes with LED stimulus lights inside. A partially-shaded house light was mounted centrally at the top of the food cup wall and provided background lighting throughout the session. Each testing chamber was enclosed in a 62.0 × 56.0 × 56.0 cm sound-attenuating chamber fitted with an exhaust fan that provided air flow to the test chamber and background white noise.

### Behavioral procedures

#### Task

The start of a session was signaled by illumination of the house light and delivery of one food pellet (45 mg, Bio-Serv). Retrieval of the pellet activated the head entry detectors at the reward port and served to initiate the first trial. Following a fixed ITI of 5 s, one stimulus port was illuminated. Animals were required to nose poke in the illuminated port to indicate a correct response. While the stimulus port was lit and for a short period afterward, termed the limited hold period, nose poke responses in the port were rewarded with delivery of a pellet. Nose poke responses in ports that were not illuminated were defined as incorrect responses. Errors of omission were defined as a failure to respond during the limited hold period. Both incorrect responses and omission errors were punished with a time out, house light off for 5 s. Nose poke responses in any of the stimulus ports or the reward port restarted the time out period. Subsequent trials were initiated by head entry to the reward port or completion of the time out period. Perseverative responses were defined as nose pokes following the initial correct response and were punished by a time out. Premature responses were defined as nose pokes made during the ITI and required the animal to initiate a trial by responding in the reward port. Daily testing sessions consisted of 100 trials or 30 min of testing, whichever occurred first. At the completion of the session the house light was extinguished and responses were no longer rewarded.

#### Shaping and training

Initially stimulus ports were illuminated for long periods to facilitate learning. At the start of shaping stimulus ports were illuminated for 64 s. Once subjects reached a criterion performance of 75% correct for two consecutive sessions, the stimulus duration was decreased to the next level. Shaping stimulus durations (limited hold) were 64, 32, 16, 8, 4, 2, 1.5, and 1 s. If subjects were not able to reach a criterion of 75% within 10 sessions, they were advanced to the next phase. Once shaping was complete, subjects completed 20 d of training in the standard condition with the 0.5-s stimulus duration.

#### Attentional challenges

Following completion of 20-d training on the standard condition, a series of behavioral challenges were introduced. Subjects completed four challenge sessions, interspersed with baseline sessions (standard condition). Challenge conditions included noise distractor presented with the target, shortened stimulus durations, variable ITIs, and tone distractor presented during the ITI. In the noise challenge, a burst of white noise was introduced immediately before illumination of the stimulus port. In the short challenge, stimulus durations were shortened from 0.5 to 0.25 s. The third challenge condition introduced variable ITIs instead of the constant 5-s ITI. Variable ITIs included 1, 3, or 5 s. Finally, in the tone challenge, a tone was introduced at random periods during the standard ITI.

### Data analysis

Shaping, training, and attentional challenge phases were analyzed separately by univariate and repeated-measures ANOVA (rANOVA). We were interested both in whether the POR and PER groups differed from the SHAM group and in whether POR and PER groups differed from each other, regardless of whether there was a main effect of lesion group. Thus, for between group analyses, we conducted planned comparisons of the POR and PER lesion groups to the SHAM group and to each other.

For analyses of shaping, stimulus duration Level was the within-subject variable. The dependent variables analyzed included sessions to criterion (STC), accuracy [number correct/(number correct + number incorrect) × 100], omissions, premature responses, perseverative responses, and head entries at the food port. In addition, we analyzed latencies for correct and incorrect responses as well as latency to respond at the food port (correct latency, incorrect latency, and reward latency).

For analysis of training, the twenty sessions were grouped into blocks of five sessions, and block was the within-subject variable. The dependent variables included accuracy, omissions, premature responses, perseverative responses, head entries, correct latency, incorrect latency, and reward latency.

For attentional challenges, challenge sessions were alternated with standard baseline sessions and performance on a challenge was compared with the prior baseline session. We first analyzed each group individually on each challenge type with Condition as the within-subject variable. We then conducted planned comparisons of the POR and PER lesion groups to the SHAM group and to each other, similar to the analyses of shaping and training except that condition was the within-subject variable rather than session or block. Finally, to better assess the responses to attentional challenges, we calculated a challenge ratio, CR = (challenge – baseline)/(challenge + baseline), such that scores near zero indicate little change from baseline during the challenge. Positive and negative scores indicate increases and decreases from baseline, respectively. The challenge ratio allows the reader to quickly see whether the challenge performance was higher or lower than baseline.

All analyses were conducted using SAS version 8.0 or 9.1 (SAS Institute) or SPSS version 24 (IBM). A significance level of 0.05 was used for all analyses.

## Results

### Histology

**Figure 1.**
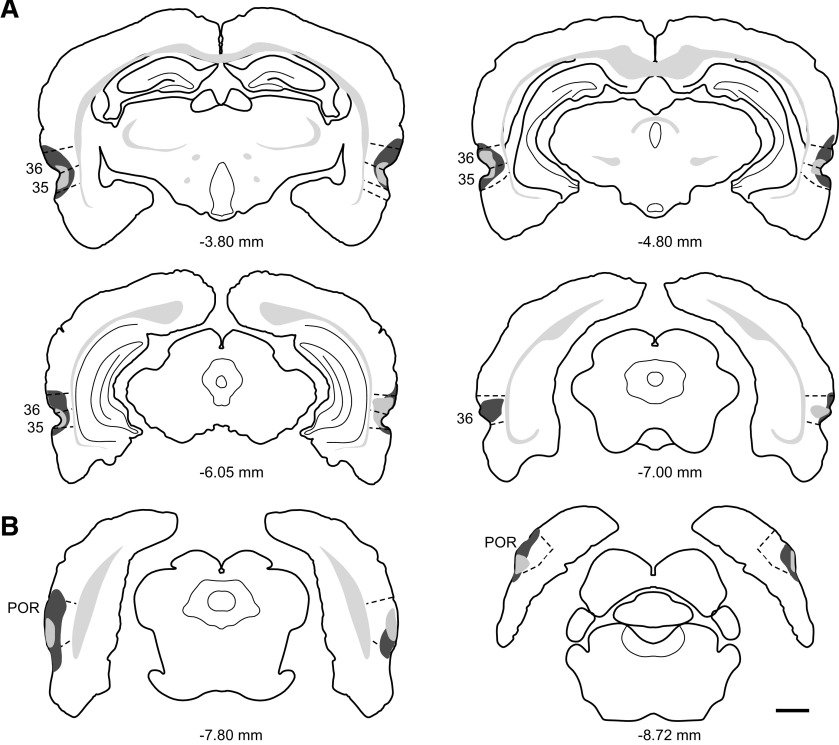
Coronal sections showing the extent of experimental lesions. Schematics of largest and smallest lesions are shown for the PER group (***A***) and the POR group (***B***). The largest lesion is shown in dark gray and the smallest lesion is shown in light gray. Scale bar: 1 mm.

Three groups of animals were tested on the 5CSRT task: POR (*n* = 8), PER (*n* = 8), and sham surgery controls (SHAM, *n* = 12). Examination of coronal sections through the POR for each lesioned subject revealed that tissue was damaged throughout the rostrocaudal extent of the region. A total of 94.5% of coronal sections exhibited some amount of damage. Using the Cavalieri method the average volume of POR damage was estimated at 36.9%. There was a very small amount of damage to the bordering medial entorhinal cortex in all POR cases, but the damage was small and predominantly unilateral. PER lesion analysis also revealed tissue damage along the entire rostrocaudal extent of the region. A total of 88.8% of coronal sections exhibited some amount of obvious damage. The average volume of PER damage was estimated at 44.9%. In seven PER cases there was some damage limited to the most lateral part of the ventrally adjacent entorhinal cortex, and in five cases there was a small amount of damage to the dorsally adjacent temporal association cortex. Schematics of the largest and smallest lesions to the POR and PER are presented in Figure 1.

**Figure 2.**
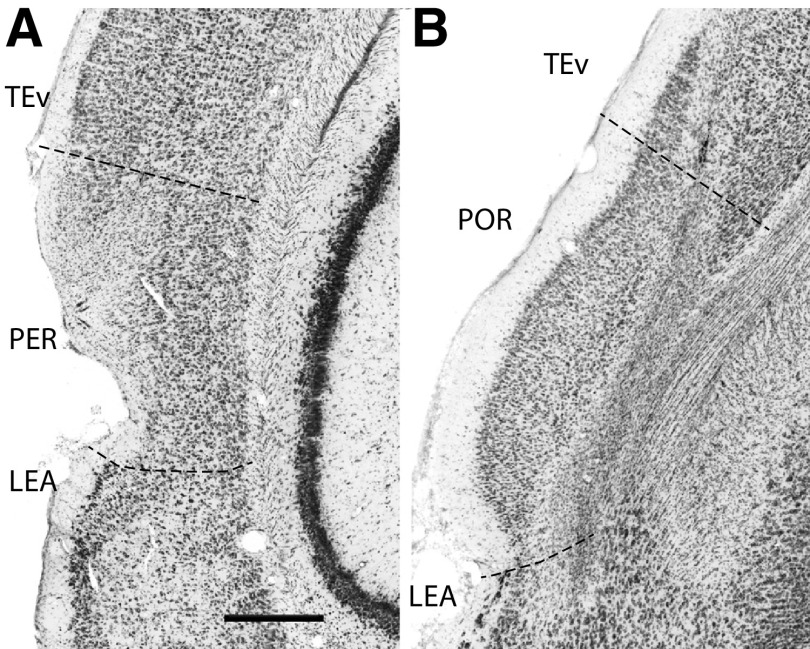
Examples of experimental lesion damage in the PER and the POR. Damage was identified as missing cortex, thinning cortex, missing cells, and pyknotic cells. ***A***, In this example from a PER subject, there was damage to the superficial layers and middle layers of the dorsoventral extent of the PER. Layer I is thinner than in control subjects. There are areas in which cells have disappeared or have become pyknotic in superficial layers and deep Layer V. ***B***, This POR subject shows damage to deep layers exhibited as pyknotic cells as well as thinning of cortical layers. Again, secondary damage would be expected in superficial layers because of the death of deep layer cells that project to superficial layers. In both cases, there is likely secondary damage that is not apparent. See text for details. In this case, there is some damage in dorsally adjacent temporal ventral cortex (TEv) and possibly to the ventrally adjacent lateral entorhinal area (LEA). In both cases, there is likely secondary damage that is not apparent. See text for details. Scale bar: 500 μm.

Figure 2 shows examples of the type of damage that that was assessed in our histologic analysis. Damage was identified as missing cortex, thinning cortex, missing cells, and pyknotic cells. Pyknotic cells appear as smaller and darker than healthy cells. One section from a PER subject (Fig. 2*A*) shows apparent damage to the superficial layers and middle layers, including superficial Layer V in the dorsoventral extent of the PER. Layer I is thinner than in control subjects. There are areas in which cells have disappeared, and there are other areas in which cells have become pyknotic. There is likely secondary damage to cells in deep layers that project to the damaged superficial layers. Additionally, the PER has intrinsic longitudinal connections which would also contribution to secondary damage not likely to be apparent in cell stains. Figure 2*B* shows a section from the brain of a POR subject. In this case, damage to deep layers presents mainly as pyknotic cells. Additionally, there is thinning of all cortical layers. Again, secondary damage would be expected in superficial layers because of dying cells in deep layers, and we would also expect that there is because of intrinsic connections. In this case, there is some damage in dorsally adjacent ventral temporal cortex. There is possibly some Layer II damage in the ventrally adjacent lateral entorhinal area, but most of the open space is actually the lamina dissecans and likely not missing cells.

Two questions of interest are (1) whether lesion size correlates with performance, and (2) whether damage outside of the target regions (PER and POR) can account for our results. To address these questions, we further analyzed our histologic data. As described in the methods, lesion size was quantified by the percentage of the region that was damaged, and lesion distribution was quantified by the percentage of coronal sections that showed any damage. Lesion size and lesion distribution were quantified separately for target region damage and for non-target region damage, yielding four variables for each subject. For POR and PER groups combined, none of the four variables was correlated with percent accuracy: *p* > 0.952 for target lesion size, *p* > 0.475 for target lesion distribution, *p* > 0.249 for non-target lesion size, and *p* > 0.149 for non-target lesion distribution. There were also no significant correlations for PER histology analyzed separately (*p*s > 0.823). For the POR analysis neither target lesion variable was significantly correlated with accuracy (*p*s > 0.549). Non-target lesion size and distribution were significantly positively correlated with accuracy for both variables. This correlation, however, was because of a single POR subject that exhibited by far the largest non-target region damage and also exhibited the highest accuracy of the group. Without that animal the POR correlation analyses were not significantly correlated with accuracy: *p* > 0.845 for non-target lesion size, and *p* > 0.907 for non-target lesion distribution. We also ran a non-parametric test, the Independent-Samples Mann–Whitney *U* test, for group differences between the PER and POR groups for all four lesion variables. There was no significant group difference on any variable: target lesion size, *p* > 0.195; target lesion distribution, *p* > 0.234: non-target lesion size, *p* > 0.798; non-target lesion distribution, *p* > 0.878. Thus, neither lesion size nor extra-target region damage can account for our results.

### Shaping

**Figure 3.**
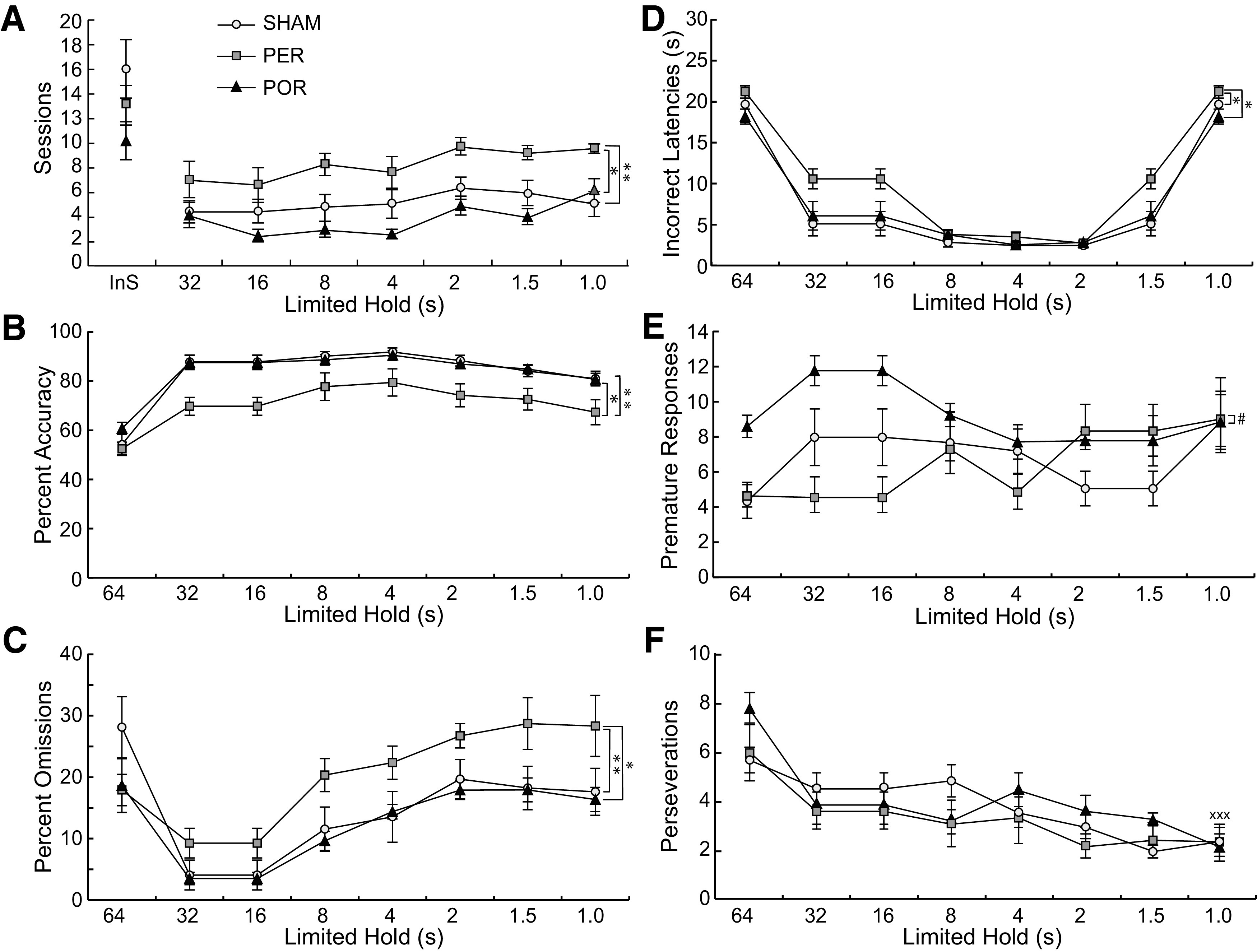
Group performance during shaping. ***A***, The PER group compared with both the SHAM and the POR groups required significantly more sessions to reach criterion. They also showed lower accuracies (***B***), higher omissions, especially in later shaping stages (***C***), and longer latencies to make an incorrect choice, especially early in shaping (***D***). ***E***, The POR group also showed marginally higher premature responses than the PER group. ***F***, The POR group differed from the SHAM group only in the pattern of perseverations, showing more perseverations at some limited holds and fewer at others. No other differences in shaping were observed. InS, initial shaping. Group differences: *p < 0.05, **p < 0.01, ***p < 0.001, #*p* < 0.075. POR versus SHAM, group by limited hold: *p* < 0.0001, xxx.

Subjects were initially tested with a stimulus duration of 64 s, and progressively shaped down to a stimulus duration of 1 s (Fig. 3*A*). A performance criterion for advancing to the next stimulus duration was set at >75% accuracy for two consecutive days. However, beginning with the 32-s duration if the criterion had not been met in 10 sessions, the subject was progressed to the next stimulus duration. All of the POR subjects reached criterion on each stimulus duration in <10 daily sessions. Seven of eight PER subjects were advanced for at least one stimulus duration. Three SHAM subjects were advanced for one or both of the shortest stimulus durations. Overall, the POR group required fewer sessions (24.00 ± 1.28) and the PER group required more sessions (55.13 ± 9.48) as compared with the SHAM group (35.85 ± 3.96) to reach criterion (Fig. 3*A*). The PER group showed significantly higher sessions to reach criterion compared with the POR group (*F*_(1,14)_ = 10.58, *p* < 0.006) and with the SHAM group (*F*_(1,17)_ = 7.37, *p* < 0.015). The POR and SHAM groups were not different from each other (*p* > 0.16). Note that because so many PER rats and a few SHAM rats were advanced without reaching criterion of 75% accuracy, their training and baseline performances tend to be lower than criterion.

In addition to slow acquisition during shaping, the PER group was impaired on a number of measures compared with the SHAM group (Fig. 3). This was evident in significant main effects of group and/or group x shaping level interactions (Fig. 3*A–C*,*F*). The PER group exhibited significantly lower percent accuracy (*F*_(1,17)_ = 9.7, *p* < 0.007; Fig. 3*B*), which improved slightly across shaping (*F*_(7,119)_ = 2.936, *p* < 0.008) and significantly higher percent omissions that worsened slightly across shaping (*F*_(7,119)_ = 2.94, *p* < 0.007; Fig. 3*C*). Finally, early in shaping, the PER group showed higher latencies to respond after incorrect trials, but latencies were similar to those of the SHAM group by the end of shaping (*F*_(7,119)_ = 3.125, *p* < 0.005; Fig. 3*D*).

The PER group was also significantly impaired on a number of measures as compared with the POR group (Fig. 3*A–F*). The PER group exhibited significantly lower percent accuracy (*F*_(1,14)_ = 8.44, *p* < 0.012; Fig. 3*B*) and significantly higher percent omissions (*F*_(1,14)_ = 12.18, *p* < 0.005; Fig. 3*C*). The PER group exhibited a trend toward fewer premature responses than the POR group (*F*_(1,14)_ = 4.15, *p* < 0.062; Fig. 3*E*). Also similar to the SHAM group, the PER group showed higher latencies to respond than the POR group after incorrect trials, but latencies were similar to those of the SHAM group by the end of shaping (*F*_(7,98)_ = 3.35, *p* < 0.031; Fig. 3*E*).

The POR and SHAM groups were not different from each other except on perseverative responses (Fig. 3*F*). There was no main effect of group but there was a group by level interaction (*F*_(7,119)_ = 4.40, *p* < 0.0003). Numerically, the POR group had more perseverations at limited holds of 64, 4, and 2 s and fewer perseverations at 32, 16, and 8 s. *Post hoc* analyses indicated that the POR group had significantly more perseverations at the 64-s level of shaping (*p* < 0.021) and marginally significantly less perseverations at the 8-s level of shaping (*p* < 0.077).

### Training

After reaching criterion during shaping, all subjects were trained on the standard task (stimulus duration of 0.5 s) for 20 d. One SHAM subject that was performing at 73.6% accuracy at the end of shaping dropped to a mean accuracy across training of 23.2%. This subject was removed from all analyses. Because of experimenter error, data files for training day 13 were lost for all but two subjects. These data were estimated for each animal by taking the mean of days 12 and 14.

**Figure 4.**
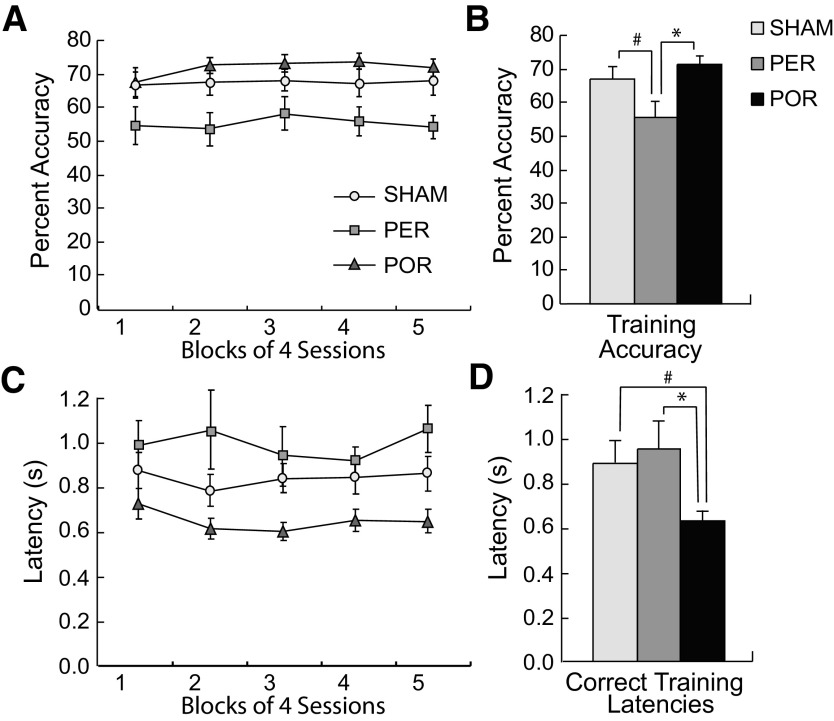
Group performance during training. ***A***, Percent accuracy across blocks of five sessions. ***B***, Overall percent accuracy. ***C***, Latencies to respond on correct trails across blocks of five sessions. ***D***, Overall latencies on correct trials. The PER group exhibited significantly lower accuracies than the POR group and marginally significantly lower accuracy than the SHAM group. The POR group exhibited significantly faster latencies on correct training trials than the PER group and marginally significantly faster latencies than the SHAM group. InS, initial shaping. Group differences: *p < 0.05, #*p* < 0.075.

Four blocks of five sessions were analyzed for the variables of interest, including accuracy, omissions, premature responses, perseverative responses, latency to respond, and latency to retrieve reward. Planned comparisons indicated no group by block interactions in performance in the standard task. There were, however, a few group differences for accuracy (Fig. 4*A*,*B*) and latencies to respond on correct trials (Fig. 4*C*,*D*). On accuracy, the two lesion groups were not different from the SHAM group but were different from each other in that the PER rats were impaired compared with the POR rats. The PER group showed marginally significantly lower accuracy compared with the SHAM group (*F*_(21,17)_ = 3.94, *p* < 0.06; Fig. 4*B*). The POR group showed similar accuracy compared with the SHAM group (*p* > 0.39) and significantly higher accuracy compared with the PER group (*F*_(1,14)_ = 8.79, *p* < 0.01). The POR group showed marginally lower latencies on correct trials compared with the SHAM group (*p* < 0.052; Fig. 4*D*) and significantly lower latencies compared with the PER group (*F*_(1,14)_ = 7.24, *p* < 0.018). Other than accuracy and latencies on correct trials, planned paired comparisons revealed no other significant between-group differences.

### Responses to attentional challenges

#### Within-subject effects

We first analyzed responses to attentional challenges for each group, individually, to determine whether there was a significant impact of each challenge on each performance measure except head entries. The measure of head entries showed very high variance. Upon inspection of the data, it was determined that the number of head entries in some session for some subjects was impossibly high. Since we were unable to determine a reasonable correction, head entries were not subjected to further analysis. One SHAM subject was inadvertently not run on the last challenge in which a tone was presented during the ITI.

Starting with the SHAM group, the Variable ITI condition in which the ITI was 1, 3, or 5 s instead of the constant 5 s, had the greatest impact on performance (Table 2, top). Accuracy was significantly lower (*F*_(1,10)_ = 10.35, *p* < 0.009), and omissions were significantly higher (*F*_(1,10)_ = 11.42, *p* < 0.007). Latencies were also significantly different with slower latencies for both correct (*F*_(1,10)_ = 5.07, *p* < 0.05) and incorrect trials (*F*_(1,10)_ = 37.26, *p* < 0.001), and faster latencies to approach the reward port (*F*_(1,10)_ = 8.16, *p* < 0.02). For the short condition (target shortened to 250 ms), accuracy was also significantly lower (*F*_(1,10)_ = 23.95, *p* < 0.001). Introducing a burst of white noise immediately before presentation of the visual target (noise condition) significantly increased omissions (*F*_(1,10)_ = 7.32, *p* < 0.02). There was no impact of tone condition (tone during the ITI) on any measure, and there was no impact of any challenge on premature responses, or perseverations.

**Table 2:** Response to challenges

Region	Noise prior to target	Short target	Variable ITI	Tone during ITI
SHAM				
Accuracy	-	↓↓↓	↓↓↓	-
Premature responses	-	-	-	-
Omissions	↑↑	-	↑↑↑	-
Perseverations	-	-	-	-
Correct latencies	-	-	↑↑	-
Incorrect latencies	-	-	↑↑↑	-
Reward latencies	-	-	↓↓	-
PER				
Accuracy	-	-	-	-
Premature responses	↑↑↑	-	↓↓	↑
Omissions	-	-	-	-
Perseverations	-	-	-	-
Correct latencies	↓	-	↑	-
Incorrect latencies	↓	-	↑↑	-
Reward latencies	-	-	-	-
POR				
Accuracy	-	-	-	-
Premature responses	-	-	-	-
Omissions	↑↑	↑	-	-
Perseverations	-	-	-	-
Correct latencies	**↑↑**	-	↑↑↑	-
Incorrect latencies	↑↑	-	↑↑↑	-
Reward latencies	↑	-	↓↓	-

Results of within subject repeated measures ANOVA in which each attentional challenge was compared with its own baseline for each lesion group. ↑↑↑*p* < 0.001, ↑↑*p* < 0.05, ↑*p* < 0.075. SHAM, controls; PER, perirhinal cortex; POR, postrhinal cortex.

**Figure 5.**
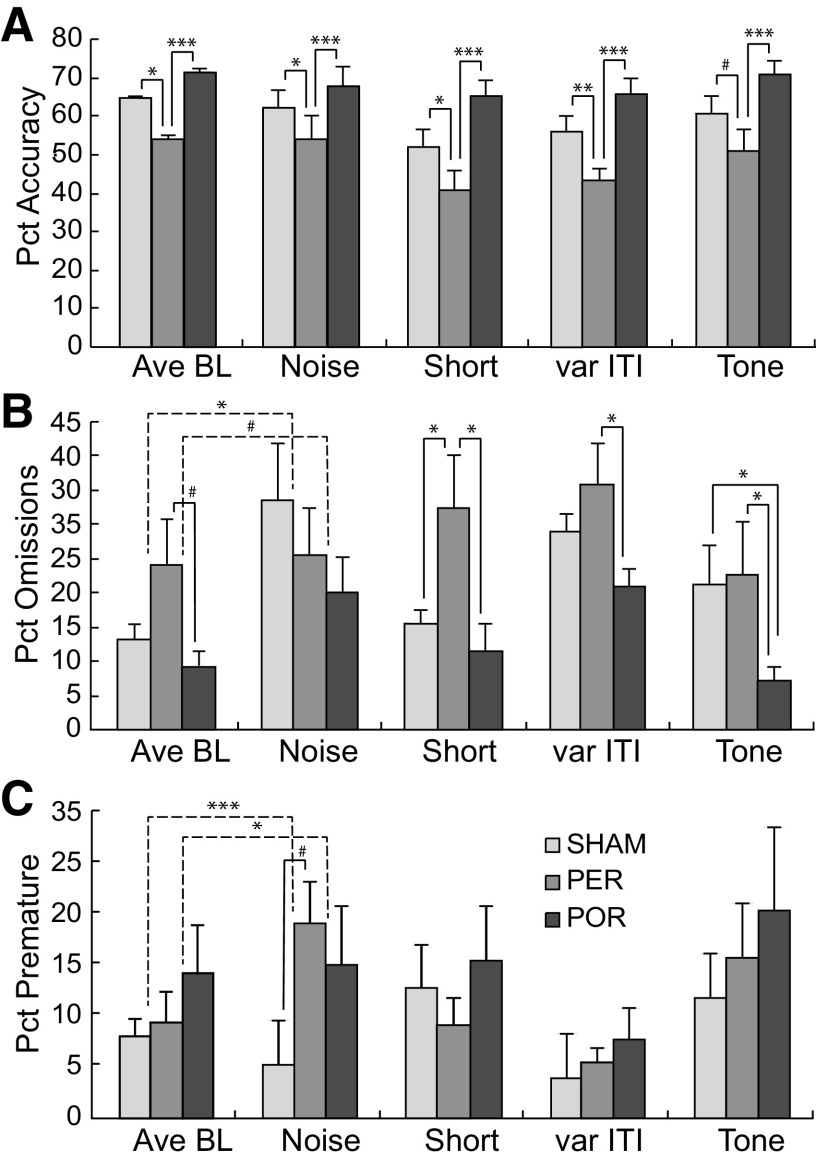
Performance during baseline and attentional challenges. Shown in each panel are the mean of the challenge baseline sessions (Ave BL) along with mean challenge performance along with group differences. Ave BL are shown for simplicity though each challenge was compared with its own baseline. It is important to note that, because there were differences in acquisition, baselines differ across groups. ***A***, For percent (Pct) accuracy, the PER group was significantly or marginally significantly lower than both SHAM and POR groups on Ave BL and each of the challenges. ***B***, For Pct omissions, the PER group was marginally significantly higher than SHAM and POR groups on baseline performance, and significantly higher than both on short challenges. The PER group was also significantly higher than the POR group n variable ITI and tone challenges. ***C***, For Pct premature responses, the ER group was significantly higher than the SHAM group on noise challenges. Group differences: *p < 0.05, **p < 0.01, ***p < 0.001, #*p* < 0.075. Solid lines indicate main effects of group and dashed lines indicate significant group by condition interactions.

**Figure 6.**
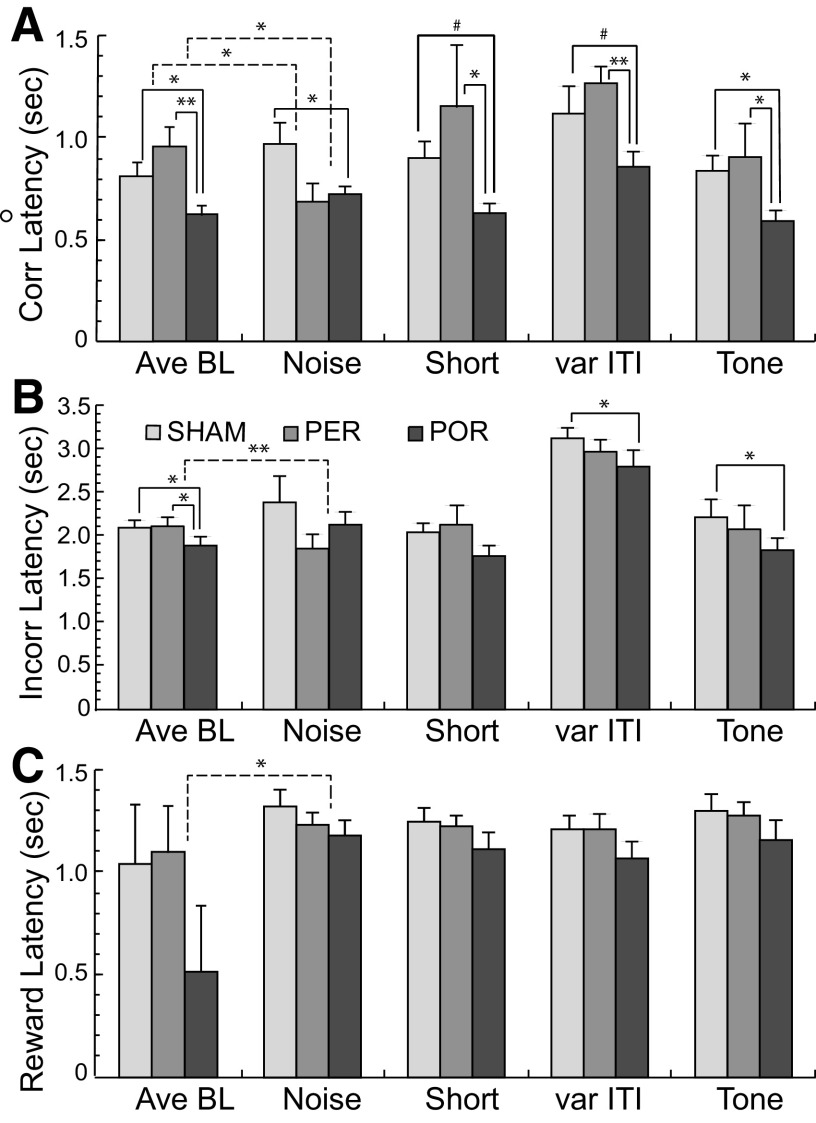
Latencies to respond during baseline and attentional challenges. Shown in each panel are the mean average baselines (Ave BL) latencies and challenge latencies along with group differences. ***A***, For correct trials, the POR group was significantly or marginally significantly faster than both SHAM and PER groups on baseline and challenges. For the noise challenge, there were group by condition interactions such that the PER group was faster relative to baseline than the POR or SHAM groups. ***B***, For latencies on incorrect trials, the POR group was significantly faster than SHAM and PER on baseline performance and the tone challenge. ***C***, For latencies to retrieve rewards there was a marginally significant group by condition interaction between the PER and POR groups for the tone challenge. The POR group was faster at baseline and the tone challenge, but the difference was less for the challenge. Significance: *p < 0.05, **p < 0.01, ***p < 0.001, #*p* < 0.075. Solid lines indicate main effects of group and dashed lines indicate significant group by condition interactions.

The PER group appeared to be less sensitive to attentional challenges than the SHAM group (Table 2, middle). Premature responses were significantly increased by the noise distractor during the target (*F*_(1,7)_ = 17.57, *p* < 0.004), significantly decreased by the variable ITI (*F*_(1,7)_ = 6.09, *p* < 0.04), and marginally significantly increased by the tone presented during the ITI (*F*_(1,7)_ = 4.99, *p* < 0.06). During the variable ITI, latencies to select were significantly higher on incorrect trials (*F*_(1,7)_ = 21.80, *p* < 0.002), and were marginally higher on correct trials (*F*_(1,7)_ = 4.68, *p* < 0.07). For the noise during target presentation challenge, latencies to select were marginally significantly higher on incorrect trials (*F*_(1,7)_ = 5.48, *p* < 0.0.052), and correct trials (*F*_(1,7)_ = 4.47, *p* < 0.072).There was no impact of the short target presentation on any measure, and there was no impact of any challenge on accuracy, omissions, perseverations, or latency to approach the reward port. It is surprising that shortening the stimulus duration did not increase premature responding, as that is a common finding in the 5CSRT task. Shortening the target duration did result in a numerical increase in premature responding for all three groups when compared with the immediately prior baseline. (Note that for ease of presentation in Figs. 5, 6, we showed the average across all baseline sessions and not the immediately prior baseline.) The variability was such that none of these increases were significant.

The POR group also appeared to be less sensitive to attentional challenges than the SHAM group (Table 2, bottom). Only omissions and latencies were affected by challenges. Omissions were significantly increased over baseline by the noise distractor during the target (*F*_(1,7)_ = 6.5, *p* < 0.04), and marginally significantly increased by the shortened target (*F*_(1,7)_ = 4.89, *p* < 0.063). For the noise during target presentation challenge, latencies to select were significantly higher on incorrect trials (*F*_(1,7)_ = 9.67, *p* < 0.0.02) and correct trials (*F*_(1,7)_ = 7.52, *p* < 0.03), and the latencies to approach the food port were marginally significantly higher on (*F*_(1,7)_ = 4.45, *p* < 0.0.073). During the variable ITI challenge, latencies to select were significantly higher on incorrect trials (*F*_(1,7)_ = 15.04, *p* < 0.0.006) and Correct trials (*F*_(1,7)_ = 19.01, *p* < 0.0.003), but the latencies to approach the food port were significantly lower (*F*_(1,7)_ = 8.84, *p* < 0.0.02). There was no impact of the tone presentation during the ITI on any measure, and there was no impact of any challenge on accuracy, premature responses, or perseverations.

#### Between-subject effects

Group differences were assessed separately for the average of the four baselines before challenge sessions and for each of the challenge sessions. Overall, there were more group differences in accuracy followed by percent omissions, and group differences were often in the same directions as for training.

For percent accuracy (Fig. 5*A*), the PER group showed significant decreases compared with the SHAM group for average baseline (*F*_(1,17)_ = 5.57, *p* < 0.03), noise (*F*_(1,17)_ = 15.34, *p* < 0.03), short (*F*_(1,17)_ = 5.04, *p* < 0.03), and variable ITI (*F*_(1,17)_ = 8.72, *p* < 0.008) conditions, and marginally significantly decreased percent accuracy for the tone condition (*F*_(1,16)_ = 3.74, *p* = 0.0710). The PER group also showed significantly decreased percent accuracy compared with the POR group (Fig. 5*A*) for the average baseline (*F*_(1,14)_ = 16.52, *p* < 0.03), noise (*F*_(1,14)_ = 9.03, *p* < 0.009), short (*F*_(1,14)_ = 15.85, *p* < 0.001), var ITI (*F*_(1,14)_ = 19.97, *p* < 0.0005), and tone (*F*_(1,14)_ = 11.06, *p* < 0.005) conditions. The POR and SHAM groups were not significantly different on accuracy of performance for baseline or for any attentional challenges.

For percent omissions (Fig. 5*B*), the PER group showed marginally significant increases compared with the SHAM group for average baseline (*F*_(1,17)_ = 3.11, *p* < 0.09) and significant increases for the short condition (*F*_(1,17)_ = 5.68, *p* < 0.03). The PER group also showed marginally significantly increased omissions compared with the POR group for the average baseline (*F*_(1,14)_ = 4.47, *p* < 0.053) and significant increases for the short (*F*_(1,14)_ = 5.63, *p* < 0.03), var ITI (*F*_(1,14)_ = 4.72, *p* < 0.04), and tone (*F*_(1,14)_ = 5.25, *p* < 0.03) conditions. The POR and SHAM groups were not significantly different on accuracy of performance for baseline or any attentional challenges. Interestingly, the POR group showed marginally significantly decreased omissions compared with the PER group for the average baseline (*F*_(1,14)_ = 4.47, *p* < 0.05) and significant decreases for the short (*F*_(1,14)_ = 5.63, *p* < 0.03), var ITI (*F*_(1,14)_ = 4.72, *p* < 0.04), and tone (*F*_(1,14)_ = 5.25, *p* < 0.03) conditions. The POR group also showed significantly decreased omissions compared with the SHAM group for the tone condition (*F*_(1,16)_ = 4.52, *p* < 0.05).

There were no significant differences for the percent trials with premature responses (Fig. 5*C*) or for percent trials with perseverative responses (data not shown). The PER group, however, showed marginally significantly increased percent premature responses compared with the SHAM group for the noise condition (*F*_(1,14)_ = 4.01, *p* < 0.065; Fig. 5*C*).

For latencies on correct trials the POR group was faster than the SHAM group on baseline and each of the challenges, and faster that the PER group on baseline and all challenges except the noise challenge (Fig. 6*A*). The POR group was significantly faster than the SHAM group on average baseline (*F*_(1,17)_ = 4.71, *p* < 0.044), the noise challenge (*F*_(1,17)_ = 7.38, *p* < 0.02), and the tone challenge (*F*_(1,16)_ = 5.86, *p* < 0.023), and was marginally significantly faster on the short (*F*_(1,17)_ = 3.96, *p* < 0.063), and variable ITI (*F*_(1,16)_ = 4.17, *p* = 0.057) challenges. The POR group also showed significantly faster latencies on correct trials compared with the PER group for average baseline (*F*_(1,14)_ = 12.79, *p* < 0.003), short (*F*_(1,14)_ = 5.62, *p* < 0.03), var ITI (*F*_(1,14)_ = 20.837, *p* < 0.0004), and tone (*F*_(1,14)_ = 7.55, *p* < 0.016) conditions. For all challenges except the noise challenge the pattern of group differences was similar to the pattern for the baseline. For the noise challenge the pattern was somewhat different. The PER group showed faster latencies compared with baseline, whereas the other two groups did not. (Fig. 6*A*). Analysis of correct latencies for the noise challenge showed significant group by condition interactions for both the PER versus SHAM comparison (*F*_(1,17)_ = 5.44, *p* < 0.032) and the PER versus POR comparison (*F*_(1,14)_ = 7.93, *p* < 0.0138).

There were fewer group differences in latencies on incorrect trials (Fig. 6*B*). The POR group was again faster than both the SHAM and the PER groups on baseline (*F*_(1,17)_ = 6.61, *p* < 0.02 and *F*_(1,14)_ = 6.77, *p* <0.021, respectively). The POR group was also significantly faster than the SHAM group on the variable ITI challenge (*F*_(1,17)_ = 6.45, *p* < 0.022), and the tone challenge (*F*_(1,16)_ = 7.09, *p* < 0.017). On the noise challenge, the POR was slower relative to baseline and PER group was faster relative to baseline. This was evident in significant group by condition interaction for the PER versus POR comparison (*F*_(1,14)_ = 14.58, *p* < 0.002).

For the latency to retrieve a food reward following a correct trial, there was only a single significant result with regard to challenges. On the noise challenge, both the POR and PER groups were slower relative to baseline, but the change was greater for the POR group (Fig. 6*C*). In other words, latencies slightly increase from baseline to noise condition for the SHAM and PER group, the latencies dramatically increased from baseline to noise condition for the POR group. This was evident in a significant group by condition interaction for the PER versus POR comparison(*F*_(1,14)_ = 6.57, *p* < 0.023).

#### Analysis of challenge ratios

In order to better assess changes from baseline during attentional challenges, we constructed a challenge ratio such that CR = (challenge – baseline)/(challenge + baseline). This ratio is mathematically equivalent for the discrimination ratio used for some novel object exploration and preferential viewing studies. Scores near zero indicate little change from baseline during the challenge. Positive scores indicate increases relative to baseline, and negative scores indicate decreases relative to baseline. We have included these analyses because they better control for baseline performance and they allow another way for the reader to assess responses to attentional challenges.

**Figure 7.**
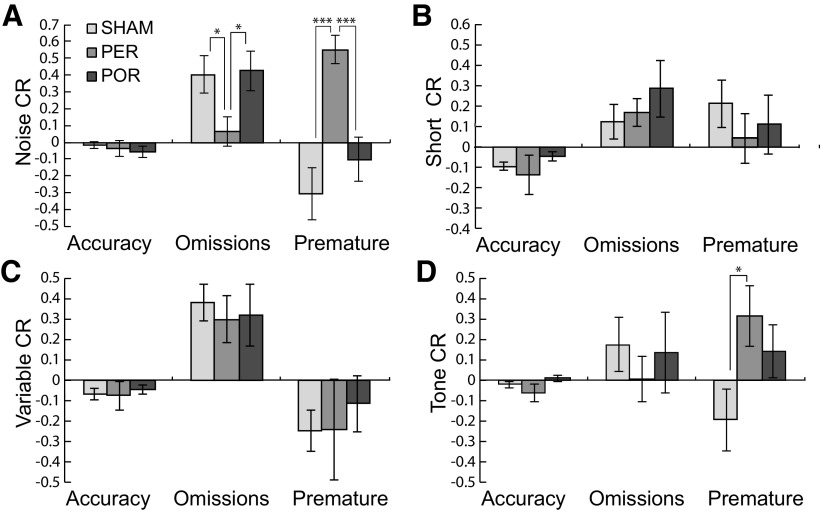
Impact of attentional challenges on accuracy, omissions, and premature responses during attentional challenges. Shown are ratios of performance on attentional challenges relative to baseline for accuracy, omitted trials, and premature responses. Differences here reflect group by condition interactions (prior baseline vs challenge). The challenge ratio is calculated as CR = (challenge – baseline)/(challenge + baseline) such that scores near zero indicate little change from baseline during the challenge. Positive and negative scores indicate increases and decreases from baseline, respectively. R is shown for the noise distraction during target presentation (***A***). Both the SHAM and POR groups showed significantly increased omissions relative to baseline. There were no significant group by condition interactions for shortened target presentations (***B***), variable ITIs (***C***), or the tone presented during the ITI (***D***). Both the SHAM and POR groups showed more omissions during the noise challenge (***A***). Group differences: *p < 0.05, ***p < 0.001, #*p* < 0.075.

ANOVA of the CR measure for group differences indicated no group differences in accuracy challenge relative to baseline, although for all groups and challenges, the mean accuracy was numerically lower on the challenge session compared with the immediately prior baseline session (Fig. 7). P values for accuracy challenge ratios ranged from 0.31 to 0.97. There were also no group differences in the ratios for perseverative responses. All perseveration ratios were close to zero, variances were high, and *p* values ranged from 0.12 to 0.75. Data are not shown for perseverations given that there were no effects on any analysis.

There were group differences in challenge ratios for omissions and premature responses for the noise and tone challenges. For the noise challenge, both the SHAM and POR groups showed increases in the ratio of challenge omissions to baseline omissions relative to the PER group (Fig. 7*A*). This was evident in significant main effects of group for SHAM versus PER (*F*_(1,17)_ = 19.303, *p* = 0.0001) and for POR versus PER (*F*_(1,14)_ = 5.827, *p* = 0.03). In contrast, the PER showed increases in the ratio of challenge premature to baseline premature responses relative to the both the SHAM and PER groups. Again, this was evident in significant main effects of group for SHAM versus PER (*F*_(1,17)_ = 19.303, *p* = 0.0001) and for POR versus PER (*F*_(1,14)_ = 17.213, *p* = 0.001). For the tone challenge (Fig. 7*D*), the PER also showed an increase in the ratio of the challenge premature to baseline premature responses relative to the SHAM and PER groups. Again, this was evident in significant main effects of group for SHAM versus PER (*F*_(1,17)_ = 19.303, *p* = 0.0001) and for POR versus PER (*F*_(1,14)_ = 17.213, *p* = 0.001).

**Figure 8.**
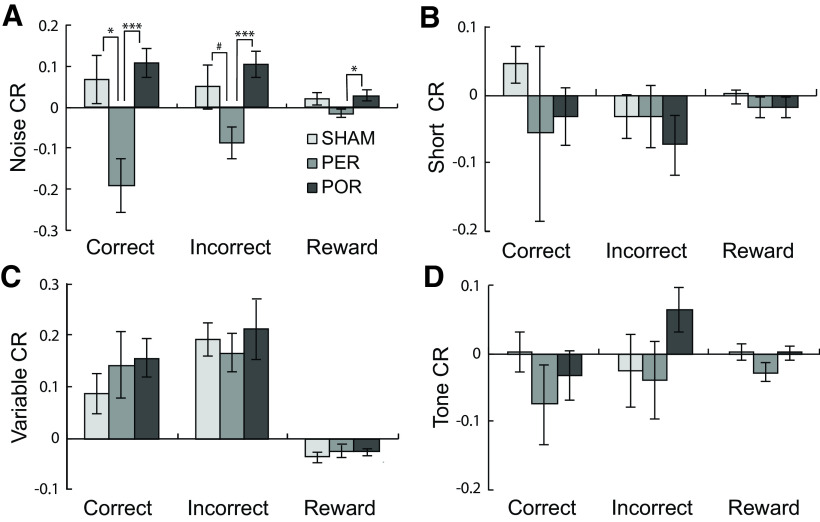
Impact of attentional challenges on latencies to respond. Shown are ratios of performance on attentional challenges relative to baseline for latencies on correct and incorrect trials as well as latencies to retrieve rewards. As in Figure 7, differences here reflect group by condition interactions. The challenge ratio is calculated as CR = (challenge – baseline)/(challenge + baseline) such that scores near zero indicate little change from baseline during the challenge. Positive and negative scores indicate increases and decreases from baseline, respectively. ***A***, For both the SHAM and POR groups, latencies changed in directions opposite that of the PER group relative to baseline. There were no significant group by condition interactions for shortened target presentations (***B***), variable ITIs (***C***), or the tone presented during the ITI (***D***). Both the SHAM and POR groups showed more omissions during the noise challenge (***A***). Group differences: *p < 0.05.

There were group differences in latency challenge ratios (Fig. 8), but only for the noise challenge. The pattern was similar for all three ratios in that the PER group became faster with the noise challenge relative to baseline and the SHAM and POR groups became slower (Fig. 8*A*). ANOVA showed significant group differences for PER versus SHAM for correct latencies (*F*_(1,17)_ = 8.278, *p* = 0.01) and marginally significant differences for incorrect latencies (*F*_(1,17)_ = 3.724, *p* = 0.07) and latency to retrieve the food reward (*F*_(1,17)_ = 3.388, *p* = 0.08). Differences were more robust for the PER versus POR comparison with significant group differences on challenge ratios for correct latencies (*F*_(1,14)_ = 15.867, *p* = 0.001), incorrect latencies (*F*_(1,17)_ = 3.724, *p* = 0.07), and latencies to retrieve the food reward (*F*_(1,14)_ = 6.776, *p* = 0.021).

## Discussion

In the present study, we used the 5CSRT task in combination with experimental lesions to address the hypothesis that the PER and POR support different types of attention. We compared rats with PER damage, POR damage, or SHAM control surgeries on acquisition, training, and attentional challenges. The results revealed a number of lesion effects among the PER, POR, and SHAM groups. During shaping, the PER group showed impaired acquisition, differing from both the POR and SHAM groups on multiple measures. During training and challenge sessions, they showed decreased accuracies compared with POR and SHAM groups. During attentional challenges, the PER rats were most affected by the presentation of noise right before target onset, and the pattern of effects suggested increased impulsivity. During the noise challenge, the PER group showed fewer omissions and more premature responses that either the POR or SHAM groups. They also showed faster latencies to respond in the noise challenge, whereas the POR and SHAM groups showed slower latencies to respond. The primary impact of POR damage during shaping was facilitated acquisition compared with the PER group. Numerically, the POR rats showed the fewest sessions to reach criterion, and unlike PER and SHAM rats, none of the POR rats were advanced from one shaping step to another without reaching criterion. During training, the POR group showed faster latencies compared with PER and SHAM groups in the absence of speed accuracy trade-off. This pattern suggests that the POR damage improves performance because of reduced monitoring of the context allowing a more specific focus on the task requirements resulting in increased attentional capacity, possibly because of better response readiness and faster processing speed.

One contribution of the present study is that it addresses a controversy about whether structures in the medial temporal lobe memory system are functionally differentiated. Prior studies suggest that some medial temporal lobe structures may have non-mnemonic functions, for example, attentional and perceptual functions (Bussey and Saksida, 2007; Murray et al., 2007; Córdova et al., 2016). The current findings provide further evidence for the view that there is functional specialization in the medial temporal lobe such that the function of some structures extends beyond the domains of learning and memory.

One limitation of our study is that the differences in acquisition make it challenging to interpret results of attentional challenges. Several PER animals and some SHAM animals were advanced to the next limited hold without meeting criterion during shaping, but no POR animals were advanced. The PER group was clearly impaired in acquisition. The PER group was at ∼67% accuracy toward the end of shaping, and the POR and SHAM groups were at ∼81%. For all three groups, accuracy decreased by ∼10% during training, possibly because we dropped the stimulus duration too steeply during the end of shaping. However, the profile was similar in that the POR and SHAM groups performed better than the PER group. Moreover, accuracy during training for all three groups was stable for 20 sessions. For these reasons, we argue that results during the training phase and during challenges provide useful and interpretable data.

During shaping, the PER group required more trials to reach criteria compared with the SHAM group. The PER group also exhibited lower accuracies and higher omissions during shaping. During training, the PER group was also significantly less accurate than the SHAM and POR groups. This profile of performance was evident in baseline and challenge performances. The PER group showed fewer changes to attentional challenges, perhaps because baseline performance was impaired and there was less parametric space for impact of PER damage on variables. Interestingly, the response of the PER group to the noise distraction challenge differed from that of the SHAM and POR groups, the PER lesioned rats showed significantly increased premature responses, generally interpreted as increased impulsivity. Since the PER exhibits strong connectivity with the medial prefrontal cortex, PER damage may have disrupted PER-medial prefrontal connections important for top-down control resulting in increased impulsivity.

This pattern of results for the PER group suggests impaired learning, decreased attentional capacity, and decreased impulse control. The impairments in learning and accuracy at asymptote could represent perceptual impairments, but is more likely attentional. The so-called perceptual deficits emerge in tasks in which animals are required to disambiguate stimuli with complex overlapping features (Bussey and Saksida, 2007; Kealy and Commins, 2011; Kent and Brown, 2012). We argue that these deficits could be described as attentional deficits. In order to disambiguate stimuli with overlapping features, animals must be able to select some features over others for further processing. Most of the discrimination work addresses the visual domain, but there is evidence for perirhinal involvement in all sensory domains including auditory (Bang and Brown, 2009), olfactory (Herzog and Otto, 1998), gustatory (Tassoni et al., 2000), and tactile (Holdstock et al., 2009) sensory domains. One possibility is that PER damage impairs the animal’s ability to disambiguate nose poke holes in the 5CSRT apparatus resulting in decreased accuracy and impaired acquisition. This could also be extended to the increased sensitivity to the noise distraction in that rats with PER damage may not have been able to disambiguate the onset of the noise from that of the visual target. However, decreased impulse control seems to be the more likely explanation for increased impulsivity when noise is presented before presentation of the visual target.

During shaping, the POR group performed similarly to the SHAM group on all measures except perseverations at the 64-s level for which the POR group showed on average, approximately two more perseverations per session. Interestingly, on correct trials during training the POR group exhibited significantly faster latencies to respond than either of the other two groups. The faster latencies for the POR group were in the absence of a decrease in accuracy, suggesting enhanced attention or greater response readiness for the POR group. The POR group was also less impacted by attentional challenges compared with the SHAM group, and the profile of responses differed particularly for the noise challenge and for the variable ITI challenge. The SHAM group showed decreased accuracies for the short and variable ITI challenges, but accuracies for the POR group were not affected by any challenge. For the noise challenge both the POR and SHAM groups showed increased omissions, but only the POR group showed longer latencies. Longer latencies were evident for the variable ITI, although unlike the SHAM group, the POR group did not show significantly decreased accuracy or increased omissions. Overall, the main impact of challenges on the POR group was slower latencies to respond with no change in accuracies, although response latencies were faster than those of the SHAM group in all conditions.

We previously provided evidence for a postrhinal role in attention, specifically attentional orienting (Bucci and Burwell, 2004). This is consistent with the anatomy in that the POR is strongly and reciprocally connected with the lateral posterior nucleus of the thalamus, which is the rodent homolog of the primate pulvinar and implicated in attention (Agster et al., 2016; Tomas Pereira et al., 2016; Kaas and Baldwin, 2019; Yang and Burwell, 2020). We have also provided evidence for the hypothesis that the POR maintains a representation of the current context and monitors the context for changes, updating the representation as changes occur (Burwell and Hafeman, 2003; Furtak et al., 2012). One possibility is that, in the absence of an intact POR, rats were not continuously monitoring the current environment and were able to focus more resources on the task resulting in better response readiness and increased attentional capacity. There is some indirect evidence for this interpretation. Earlier we showed that rats with combined lesions of the PER and POR trained on a complex feature positive/feature negative discrimination (FPFN) task showed facilitated acquisition (Gastelum et al., 2012). We interpreted the facilitation as resulting from POR damage, because rats with PER damage are impaired on the feature-negative task (LT1, T2) and on positive patterning (LT1, L2, T2), when the compounds are presented simultaneously (Campolattaro and Freeman, 2006a,b). Additionally, in the FPFN task, context is thought to accrue inhibitory control over other cues. Without context representations this inhibitory control would fail, and animals would be expected to learn the task more efficiently. Accordingly, our interpretation is that the POR damage impaired contextual control resulting in facilitated attentional monitoring. Thus, the present findings provide more direct evidence for a POR role in stimulus driven, automatic attention.

How might PER and POR attentional functions be used? We have suggested that the PER and POR support different types of attention in the service of learning and memory. There is anatomic evidence for this assertion given that the PER and POR provide direct inputs to the entorhinal cortex, subiculum, CA1, presubiculum, and parasubiculum (Agster and Burwell, 2013). Indeed, an open question about episodic memory is how items to be remembered are selected from all the possibilities available at any given time. Regions outside the medial temporal lobe that connect with the PER and POR/PHC have been proposed to serve this function (Córdova et al., 2016). Here, we provide evidence that the PER and POR/PHC, themselves, are important for the selection of information to be remembered. It may also be the case that the PER and POR support attentional and executive functions of other brain regions. For example, the PER is strongly connected with medial prefrontal cortex, whereas the POR is strongly connected with ventrolateral orbital prefrontal cortex. Based on functional and anatomic evidence, we have suggested the PER and POR cortices act as a contextual-support network that directly provides contextual and spatial information to the prefrontal cortex (Peng and Burwell, 2021). In addition to its robust connections with the PER, the POR is also strongly interconnected with a number of visuospatial processing regions including the posterior parietal cortex, retrosplenial cortex, visual association cortex, and the pulvinar (Agster and Burwell, 2009, 2013; Agster et al., 2016; Tomas Pereira et al., 2016; Yang et al., 2021). Such connectivity could support the POR binding of non-spatial information from the PER with spatial information to represent the current local physical context, including the spatial layout of objects and features in the environment as well as the geometry of the space. The POR then automatically monitors the environment for changes and updates representations when changes occur. These representations of context are available to be used by multiple brain regions, including prefrontal, posterior cortical, and hippocampal areas, for context-guided behavior, associative learning, and episodic memory (Peng and Burwell, 2021).

In summary, the PER and POR, structures in the medial temporal lobe, are implicated in learning and memory. Whether medial temporal lobe structures are exclusively dedicated to memory, however, is under debate. Here, we provide evidence that the functions of the PER and POR cortices extend beyond memory for objects and spatial memory for include attentional functions. Specifically, the PER contributes to controlled attention, and the POR contributes to stimulus-driven attention. These findings provide new evidence for functional specialization in the medial temporal lobe.

## Synthesis

Reviewing Editor: Karyn Frick, University of Wisconsin-Milwaukee Decisions are customarily a result of the Reviewing Editor and the peer reviewers coming together and discussing their recommendations until a consensus is reached. When revisions are invited, a fact-based synthesis statement explaining their decision and outlining what is needed to prepare a revision will be listed below. The following reviewer(s) agreed to reveal their identity: NONE. Note: If this manuscript was transferred from JNeurosci and a decision was made to accept the manuscript without peer review, a brief statement to this effect will instead be what is listed below.

This paper is a revision of a paper originally submitted to the Journal of Neuroscience. The reviewers found the study thoughtful, well-controlled and methodologically sound, and well written, especially in light of the complicated phenomena examined. The authors have adequately addressed the reviewer's critiques, and so I am recommending acceptance to eNeuro as is.
